# Artesunate preserves post-resuscitation myocardial and neurologic function in a rat model of cardiac arrest and cardiopulmonary resuscitation

**DOI:** 10.1016/j.resplu.2025.101214

**Published:** 2025-12-30

**Authors:** Hui Li, Cheng Cheng, Lian Liang, Tao Jin, Guozhen Zhang, Mary Ann Peberdy, Joseph P. Ornato, Wanchun Tang, Min Yang

**Affiliations:** a The Second Department of Critical Care Medicine, The Second Affiliated Hospital of Anhui Medical University, 678 Furong Road, 230601 Hefei, Anhui Province, China; b Weil Institute of Emergency and Critical Care Research, Richmond, VA, USA; c Department of Cardiology, The First Hospital of Anhui Medical University, Hefei, China; d Departments of Internal Medicine and Emergency Medicine, Virginia Commonwealth University Health System, Richmond, VA, USA; e Department of Emergency Medicine, Virginia Commonwealth University Health System, Richmond, VA, USA

**Keywords:** Post-resuscitation, Myocardial function, Neurologic function, Artesunate, Oxidative stress

## Abstract

**Background:**

To investigate the effects of Artesunate (Art) on post-resuscitation myocardial and neurologic function, survival duration, and the underlying mechanisms in a rat model of cardiac arrest (CA) and cardiopulmonary resuscitation (CPR).

**Methods:**

Thirty healthy male Sprague-Dawley rats were randomly allocated into three groups: Sham, Control(CA/CPR + vehicle),and ART(CA/CPR + Art). The latter two groups were further divided into survival and non-survival subgroups. CA were induced via 6-minute ventricular fibrillation, followed by 8 min of CPR. After the return of spontaneous circulation (ROSC), rats in the respective groups received either a vehicle or Art injection at random. Electrocardiogram (ECG) and arterial pressure were continuously monitored. In non-survival subgroups (euthanized 4 h post-ROSC), serum and tissue samples were analyzed for inflammatory cytokine concentrations, oxidative stress indices, myocardial injury markers, phosphorylated p38 (pp38), and echocardiography was performed. In survival subgroups, neurological deficit scores (NDS) were assessed at 24, 48, and 72 h post-ROSC, along with monitoring the duration of survival.

**Results:**

Art reduced the severity of post-resuscitation myocardial dysfunction compared to control group. It attenuated interleukin-6 (IL-6), tumor necrosis factor-α (TNF-α), and cardiac troponin I (cTnI) plasma levels 4 h after ROSC. In comparison with the control group, Art treatment led to a marked decrease in Thiobarbituric acid reactive species (TBARS) and 4-hydroxy-2-nonenal(4-HNE) expression, accompanied by upregulation of superoxide dismutases (SOD) activity in both heart and brain tissues. Art administration also downregulated the phosphorylation of p38. Post-resuscitation neurologic function and duration of survival were improved significantly in Art treated animals.

**Conclusions:**

This study demonstrated that Art reduces the severity of post-resuscitation myocardial and neurologic dysfunction, improves survival duration in a rat model of CA. The underlying mechanism may be related to anti-inflammation, oxidative stress and may be associated with regulation of 4-HNE induced p38 Mitogen activated protein kinase (MAPK) pathway activation.

## Introduction

Sudden cardiac arrest (SCA) is one of the leading causes of death worldwide.^1^, ^2^ Despite advances in resuscitation techniques and post-resuscitation care, the prognosis for SCA remain poor. Survival rates for out-of-hospital cardiac arrest are estimated at 7.5–15 %, while in-hospital cardiac arrest survival rates can reach up to 23.6 %.^3^, ^4^ The mechanisms underlying the poor prognosis in SCA patients remain incompletely understood. The poor prognosis associated with SCA is primarily attributed to post-cardiac arrest syndrome (PCAS). PCAS is a complex and multifaceted condition characterized by a cascade of pathophysiological events, especially post-resuscitation myocardial and neurological injury. These clinical manifestations are closely linked to systemic ischemia–reperfusion injury (IRI), which occurs when blood flow is restored to tissues that were previously ischemic. IRI triggers a robust systemic inflammatory response that leads to further tissue damage and organ dysfunction.^5^, ^6^ At present, the primary focus of PCAS management is on supportive care measures, such as optimizing hemodynamics, maintaining adequate oxygenation, and controlling body temperature.^2^

However, effective pharmacological strategies specifically targeting post-resuscitation IRI and the associated inflammatory and oxidative stress responses are still lacking. Artemisinin is isolated from the medicinal herb *Artemisia annua* L.^7^, ^8^ Its semi-synthetic derivative, artesunate (Art), has been widely adopted globally as a first-line treatment for severe malaria.^9^, ^10^, ^11^ In addition to its antimalarial effects, emerging evidence suggests that Art possesses a diverse range of biological activities, including immune-regulatory, anti-inflammatory, and antioxidant properties.^12^, ^13^, ^14^, ^15^, ^16^

Furthermore, basic research has provided solid evidence indicating that Art can effectively alleviate IRI in organs.^17^, ^18^, ^19^ Further exploration of its mechanism of action has revealed that Art can exert protective effects through multiple pathways, such as by regulating oxidative stress and mitophagy.^20^, ^21^ In the cardiac arrest and cardiopulmonary resuscitation (CA/CPR) model, myocardial and neurological damage caused by PCAS is closely linked to complex pathological changes induced by IRI.^6^ During IRI, cells undergo a series of stress responses that lead to tissue and organ damage. This is also the primary cause of organ dysfunction after cardiac arrest.^22^ Due to its multi-pathway mechanism of action, Art has the potential to intervene in this pathological process. Art may be able to regulate the various adverse effects triggered by IRI and protect the myocardium and nerves in the CA/CPR model. Ultimately, Art can improve organ function after resuscitation and enhance the survival probability.

Due to the absence of effective PCAS treatments and the existence of encouraging preclinical data on Art, this study evaluates the effects of Art on cardiac and neurological functions in a rat CA/CPR model. The study also explores the mechanisms by which Art regulates inflammation and oxidative stress. The aim is to clarify Art’s therapeutic potential for SCA survivors and provide a solid basis for future trials.

## Materials and methods

This study was performed under a protocol (AD10001396, specific aim 21) approved by the Institutional Animal Care and Use Committee of Virginia Commonwealth University. All animal experiments were conducted in accordance with the National Institutes of Health *Guide for the Care and Use of Laboratory Animals* (National Research Council, 8th edition, 2011).

Thirty male Sprague–Dawley rats (6–8 months old, 450–550 g) were supplied by a single-source breeder (Envigo, Frederick, MD). Only male rats were used in this study to reduce biological variability related to the estrous cycle and sex hormone fluctuations. Female rodents exhibit cyclical changes in estrogen levels, which may affect experimental outcomes. Using male rats with more stable hormone levels helped to obtain more consistent data in this initial mechanistic study. Animals were housed under standard laboratory conditions with free access to food and water, and maintained on a 12-h light/dark cycle. After the rats were anesthetized with CO_2_ inhalation for 30 s, the animals were anesthetized by pentobarbital (45 mg/kg intraperitoneal injection, maintained using 10 mg/kg intraperitoneal injection). Anesthetic depth was assessed by the absence of a withdrawal response to tail pinch. The trachea was intubated orally with a 14G cannula mounted on a blunt needle (Abbocath‐T; Abbott Hospital Products Division, North Chicago, IL) with an angled tip at 145°. End-tidal carbon dioxide (ETCO_2_) was continuously monitored with a side‐stream infrared CO_2_ analyzer (Capstar‐100 Carbon Dioxide Analyzer; CWE, Ardmore, PA), which was placed between the tracheal cannula and ventilator. A conventional lead II Electrocardiogram (ECG) was monitored continuously. A PE‐50 catheter (Becton Dickinson, Sparks, MD) was advanced into the right atrium through the left external jugular vein to measure right atrial pressures. A 3F catheter (Model C‐PMS‐301J; Cook Critical Care, Bloomington, IN) was advanced through the left external jugular vein into the right ventricle to induce ventricular fibrillation (VF). A thermocouple microprobe (IT-18; Physitemp Instruments Inc, Clifton, NJ) was inserted into the left femoral vein to measure blood temperature. To measure blood pressure within the descending aorta and administer Art or phosphate buffer, PE‐50 catheters were advanced into the descending aorta from the left femoral artery and the inferior vena cava from the right femoral vein, respectively. A guidewire was then advanced into the right ventricle to induce VF. The position of the guidewire was confirmed by an endocardial electrocardiogram. All catheters were flushed intermittently with saline containing 2.5 IU/mL of crystalline bovine heparin. During the experiment, the blood temperature was maintained at 37 ± 0.5 °C using a warming surgical board.

### Experimental procedures

A total of 30 rats were randomized into 3 groups using Sealed Envelope Method: a Sham group (*n* = 6), a Control group (*n* = 12), and an ART group (*n* = 12). In the Control and ART groups, half of the rats (*n* = 6 per group) were further randomized by Sealed Envelope Method to undergo the mechanism study, the remaining half were allocated for survival analysis. The Art (U.S. Pharmacopeia Company, Rockville, MD, USA) was diluted in a phosphate buffer solution (vehicle; NaH_2_PO_4_ 18.3 mM; Na_2_HPO_4_ 150.4 mM; pH 7.9–8.1). For the Control and ART groups, either the vehicle (1 mL/kg) or the Art (2.4 mg/mL/kg) was administered intravenously at the time of return of spontaneous circulation (ROSC). The investigators were blinded to group randomization.

Baseline measurements, echocardiography, and sublingual microcirculation were accomplished fifteen minutes before induction of VF. Mechanical ventilation was performed with a tidal volume of 0.6 mL/100 g of body weight, a frequency of 100 breaths/min, and a fraction of inspired oxygen (FiO_2_) of 0.21. VF was then induced through a guide wire advanced into the right ventricle. A progressive increase in 60‐Hz current up to a maximum of 3.5 mA was delivered to the right ventricular endocardium. Mechanical ventilation was discontinued after the onset of VF. The current was continued for 3 min to prevent spontaneous defibrillation. After 6 min of untreated VF, precordial chest (PC) compression and mechanical ventilation (tidal volume 0.6 mL/100 g body weight, frequency 100 breaths/min, fraction of inspired O_2_ 1.0) were initiated using a pneumatically driven mechanical chest compressor. PC was maintained at a rate of 200/min, synchronized to provide a compression-to-ventilation ratio of 2:1, with equal compression and relaxation, for 8 min. Defibrillation was attempted with up to 3 4-J countershocks after 8 min of CPR. If ROSC, defined as mean aortic pressure above 50 mmHg for 5 min, was not achieved after the first defibrillation attempt, a 30‐s interval of CPR was performed before the next defibrillation attempt (up to 3 attempts). After ROSC was achieved, the fraction of inspired oxygen was maintained at 1.0 for 1 h, then adjusted to 0.5 for the second hour and 0.21 thereafter. Rats in the Sham group underwent the surgical procedure without VF and CPR, and then followed the same intensive care program as VF rats for 4 h after ROSC. After ROSC, rats in the mechanism study group (both Control and ART) were observed for 4 h. At 4 h after ROSC, rats in the non-survival subgroup were euthanized by an intravenous injection of Euthasol (A commercial euthanasia solution containing pentobarbital sodium and phenytoin sodium, 150 mg/kg), and heart and brain tissues were immediately harvested and frozen in liquid nitrogen for further assay. Other rats in the survival analysis group received a single subcutaneous injection of buprenorphine (1 mg/kg), were returned to their cages with food and water, and were observed for 72 h. At the end of the observation period, all surviving animals were euthanized with an intravenous injection of Euthasol (150 mg/kg) under deep anesthesia.

### Measurements

ECG, mean aortic pressures (MAP), right atrial pressures, ETCO_2_, and core temperature values were continuously recorded on a personal computer–based data acquisition system supported by WINDAQ software (DATAQ, Akron, Ohio). Coronary perfusion pressure (CPP) was calculated as the difference between time‐coincident diastolic aortic and right atrial pressures displayed in real time. Sublingual microcirculation was measured at baseline and 1, 2, 3, and 4 h after ROSC using a side‐stream dark‐field imaging device (MicroScan; Microvision Medical Inc., Amsterdam, the Netherlands). The microcirculatory flow index (MFI) was measured using the method of Spronk et al.^23^ The image was divided into 4 quadrants, and the predominant type of flow (absent = 0, intermittent = 1, sluggish = 2, normal = 3) was assessed in the small vessels (<20 μm in diameter) of each quadrant. The MFI score represented the average values of the 4 quadrants. Perfused vessel density (PVD) was quantitated based on the method of De Backer et al.^24^ Vessel density was calculated by dividing the number of vessels crossing the lines by the total length of the lines. Myocardial function, including cardiac output (CO), ejection fraction (EF), and the myocardial performance index (MPI), was measured at baseline and every hour after ROSC using echocardiography (HD11XE; Philips Medical Systems, Eindhoven, the Netherlands) with a 12.5‐Hz transducer. CO and EF were used to estimate myocardial contractility; MPI was used to estimate left ventricular diastolic function. MPI is the sum of isovolumic contraction time and isovolumic relaxation time divided by ejection time. MPI was derived as (a − b)/b, where “a” is the measured duration from mitral closure to opening, and “b” is the aortic flow ejection time. Levels of consciousness, brainstem function, and overall performance were used to evaluate neurologic function according to the method of Neurologic Deficit Score (NDS), which ranged from 0 (no observed neurologic deficit) to 500 (death or brain death).^25^ The NDS was examined at 24, 48, and 72 h after ROSC.

Homogenized blood samples were collected at baseline and at the end of the observation period from different groups. Supernatants were isolated after 3000*g* at 4 °C, the resulting supernatants were analyzed for plasma levels of interleukin-6 (IL-6), tumor necrosis factor-alpha (TNF-α), and cardiac troponin I (cTnI) levels using an ELISA kit specific for rats (R&D System, Minneapolis, MN) according to the manufacturer’s instructions. Absorbance values were analyzed at 450 nm using an iMark Microplate Reader (Bio-Rad Laboratories, Inc. Hercules, CA).

Heart and brain tissue were obtained from different groups and divided into 4 sections 4 h after ROSC. The tissue was then stored at −80 °C for thiobarbituric acid reactive species (TBARS), superoxide dismutase (SOD) and Western blot analyses. The tissues was homogenized in lysis buffer and then centrifuged. TBARS and SOD levels in the supernatant were determined using SOD (Item No. 706002, Cayman Chemical Company, Ann Arbor, MI) and TBARS (Item No. 10009055, Cayman Chemical Company, Ann Arbor, MI) assay kits. All measurements were performed according to the manufacturers' instructions.

Total proteins were acquired from tissue samples by RIPA lysis buffer (ab156034, Abcam, Cambridge, UK) containing fresh protease inhibitor cocktail (P8340, Sigma-Aldrich, Merck KGaA, Darmstadt, Germany) and Phosphatase Inhibitor Cocktail (P5726, Sigma-Aldrich, Merck KGaA, Darmstadt, Germany). Supernatant containing the extracted protein were isolated after 12,000*g* for 20 min at 4 °C. The protein concentration was measured using DC protein assay kit 1 (5000111, Bio-Rad Laboratories, Inc. Hercules, CA). Equal amounts of protein (50 µg) will be separated by mini PROTEAN TGX Stain-Free Precast Gel (4568096, Bio-Rad Laboratories, Inc. Hercules, CA), then transferred onto polyvinylidene difluoride membranes (Bio-Rad Laboratories, Inc. Hercules, CA) and then assessed by ChemiDoc MP Imaging System (Bio-Rad Laboratories, Inc. Hercules, CA) Ponceau S solution staining. Membranes will be blocked with in TBS/0.1 % Casein Blocker for 1 h at room temperature and then incubated overnight at 4 °C with primary antibodies specific to phosphorylated p38 (pp38, Cat no. 9211s; 1:500; Cell Signaling Technology, Inc, Danvers, MA), p38 (Cat no. 9212s; 1:1000; Cell Signaling Technology, Inc, Danvers, MA), 4-hydroxy-2-nonenal(4-HNE, ab46545, 1:1000, Abcam, Cambridge, UK). Blots will be washed three times in TBST buffer and subsequently incubated with horseradish peroxidase-conjugated anti-rabbit secondary antibody (ab205718, 1:5000, Abcam, Cambridge, UK) at room temperature for 1 h. Bands were visualized and analyzed by means of ChemiDoc MP Imaging System (Bio-Rad Laboratories, Inc. Hercules, CA). The GAPDH protein served as an internal control (1:5000, Abcam, Cambridge, UK).

### Statistical analysis

Sample size was calculated based on our previous study to detect a difference with respect to EF (primary outcome) of ≥40 %, using G*Power software with an alpha of 0.05 and 80 %.^26^, ^27^ Potential confounders related to procedural order were minimized by randomization. Behavioral testing was performed in a randomized order across experimental days. However, cage location within the rack was not systematically controlled. Due to the nature of the intervention, the personnel performing the surgeries were aware of the group allocation. Investigators performing outcome measurements and related analyses were blinded to group allocation to support unbiased assessment. Quantitative data were reported as the mean ± standard deviation (SD), and statistics analyses were performed using SPSS 25.0 software (SPSS Inc., Chicago, IL). One-way analysis of variance (ANOVA) was used to analyze differences between groups. Comparisons of time-based measurements within each group were performed using repeated-measures ANOVA. The survival rate was calculated using Gehan-Breslow-Wilcoxon test. A *p* value of <0.05 was considered statistically significant.

## Results

### Baseline parameters

At Baseline, there were no significant differences in hemodynamics, blood temperature, myocardial function (including EF, CO and MPI), or sublingual microcirculation parameters (including MFI and PVD) among the Sham, Control, and ART groups (*P* > 0.05) (Supplementary Table S1).

### Art effectively mitigates post-resuscitation myocardial dysfunction

**Fig. 1:**
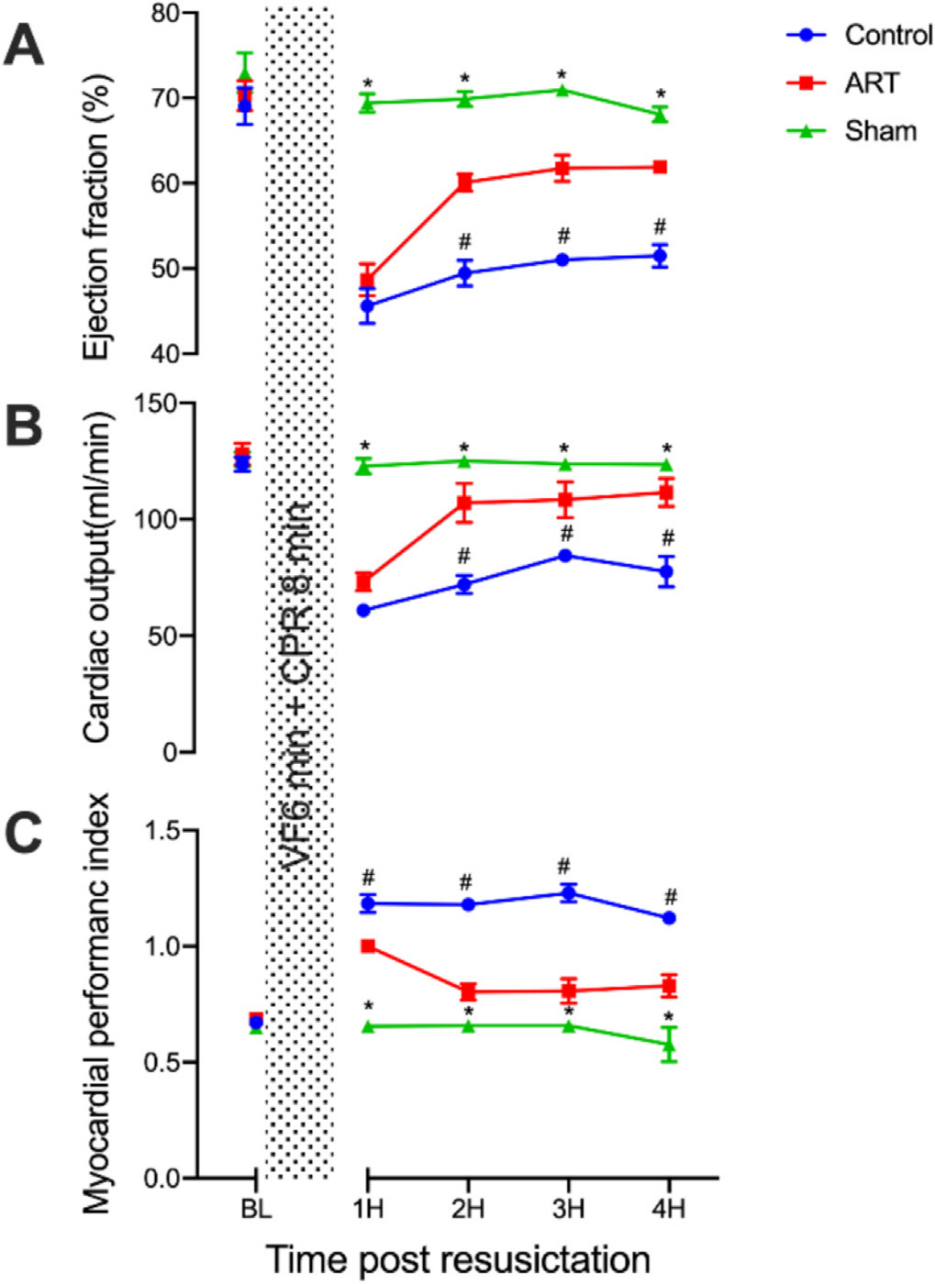
**Art improves post-resuscitation myocardial function**. Ejection fraction, cardiac output and myocardial performance index were measured by echocardiography. BL, baseline; H, hour; VF, ventricular fibrillation; CA: Cardiac arrest; CPR, cardiopulmonary resuscitation. Sham, surgical sham group without cardiac arrest; Control, CA/CPR + vehicle non-survival group; ART, CA/CPR + Art non-survival group. Data were presented as mean ± SD (*n* = 6 in each group). 2H–4H in A and C, 2H and 4H in B, all ^#^*p* < 0.0001 versus Sham; 3H in B, ^#^*p* = 0.0012 versus ART; 1H in C ^#^*p* = 0.0011 versus ART.

During CPR, there were no significant differences in CPP between the Control and ART groups. However, after resuscitation, while both groups showed a significant decline in myocardial function parameters (EF, CO, and MPI) compared to baseline, the severity of post-resuscitation myocardial dysfunction was significantly reduced in the ART group compared to the control group (Fig. 1, Supplementary Table S2), suggesting that Art attenuates myocardial dysfunction following resuscitation. Representative echocardiographic images used for the measurement of EF, CO, and MPI were shown in Fig. S1.

### Art significantly alleviates post-resuscitation microcirculatory disturbances

**Fig. 2:**
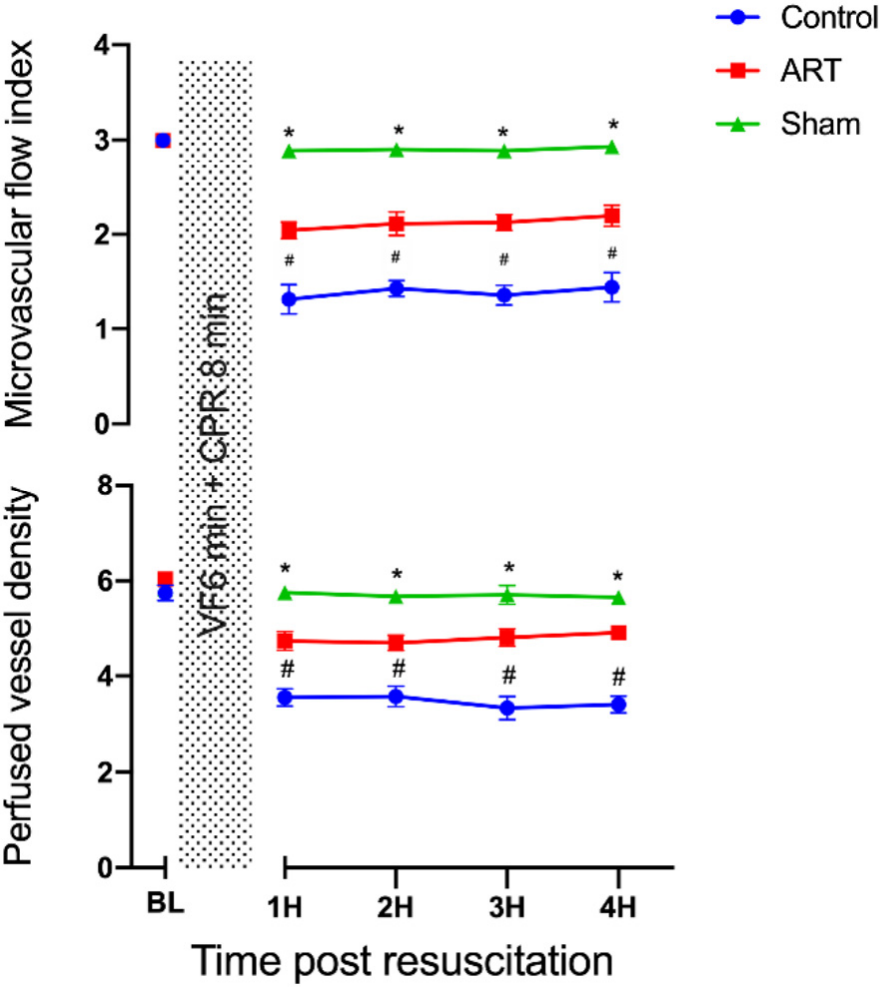
**Art improves post-resuscitation sublingual microcirculation**. Sublingual microcirculation was measured by using side-stream dark-field imaging device. BL, baseline; H, hour; VF, ventricular fibrillation; CA: Cardiac arrest, CPR, cardiopulmonary resuscitation. Sham, surgical sham group without cardiac arrest; Control, CA/CPR + vehicle non-survival group; ART, CA/CPR + Art non-survival group. Data were presented as mean ± SD (*n* = 6 in each group). All *p < 0.0001 versus Sham, All ^#^*p* < 0.0001 versus ART.

Post-resuscitation, both groups exhibited a significant reduction in sublingual microcirculation parameters (MFI and PVD) compared to baseline values (Fig. 2). Notably, the severity of post-resuscitation microcirculation dysfunction was significantly less pronounced in animals treated with Art compared to those in the control group (Fig. 2, Supplementary Table S2), indicating that Art mitigates microcirculatory disturbances after resuscitation. As shown in Fig. S1, representative sublingual microcirculation images from the Sham, Control and ART group were presented (Fig. S2).

### Art effectively prolongs survival time in experimental animals

**Fig. 3:**
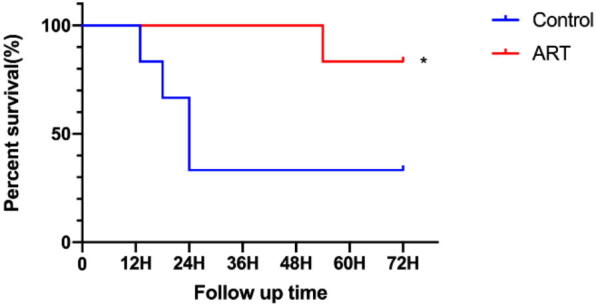
**Art improves post-resuscitation survival rate**. Survival time and the number of surviving animals were used to construct the survival curves. H, hour; Control, CA/CPR + vehicle survival group; ART, CA/CPR + Art survival group. Data were presented as mean ± SD (*n* = 6 in each group). *p = 0.045 versus Control.

The Control group exhibited a significantly shorter survival duration compared to the ART group (Fig. 3, Supplementary Table S2). Specifically, in the Control group, four rats survived for 24 h, and only two rats survived for 72 h. In contrast, all rats in the ART group survived for 48 h, and five rats survived for 72 h, demonstrating that Art prolongs survival time post-resuscitation.

### Art significantly reduces neurological deficit scores

**Fig. 4:**
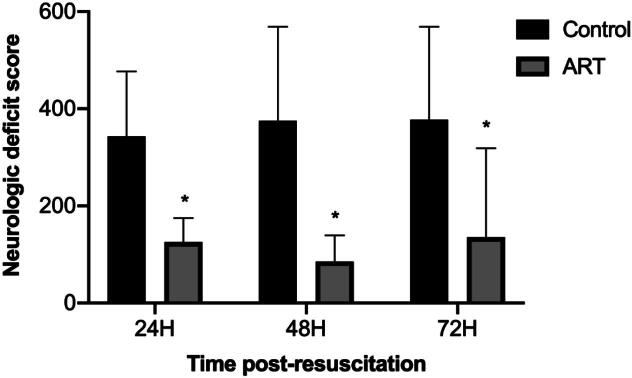
**Art improves post-resuscitation neurologic function**. Neurologic deficit score (NDS) was used to evaluate neurologic function. H, hour. Control, CA/CPR + vehicle survival group; ART, CA/CPR + Art survival group. Data were presented as mean ± SD (*n* = 6 in each group). 24H *p = 0.047 versus Control, 48H *p = 0.006 versus Control, 78H *p = 0.025 versus Control.

NDS (with a maximum deficit score of 500) were assessed daily after resuscitation. Rats treated with Art showed significantly lower NDS values at all time points post-resuscitation compared to those in the Control group (Fig. 4, Supplementary Table S2), suggesting that Art reduces neurological deficits following resuscitation.

### Art lowers levels of inflammatory and myocardial injury markers

**Fig. 5:**
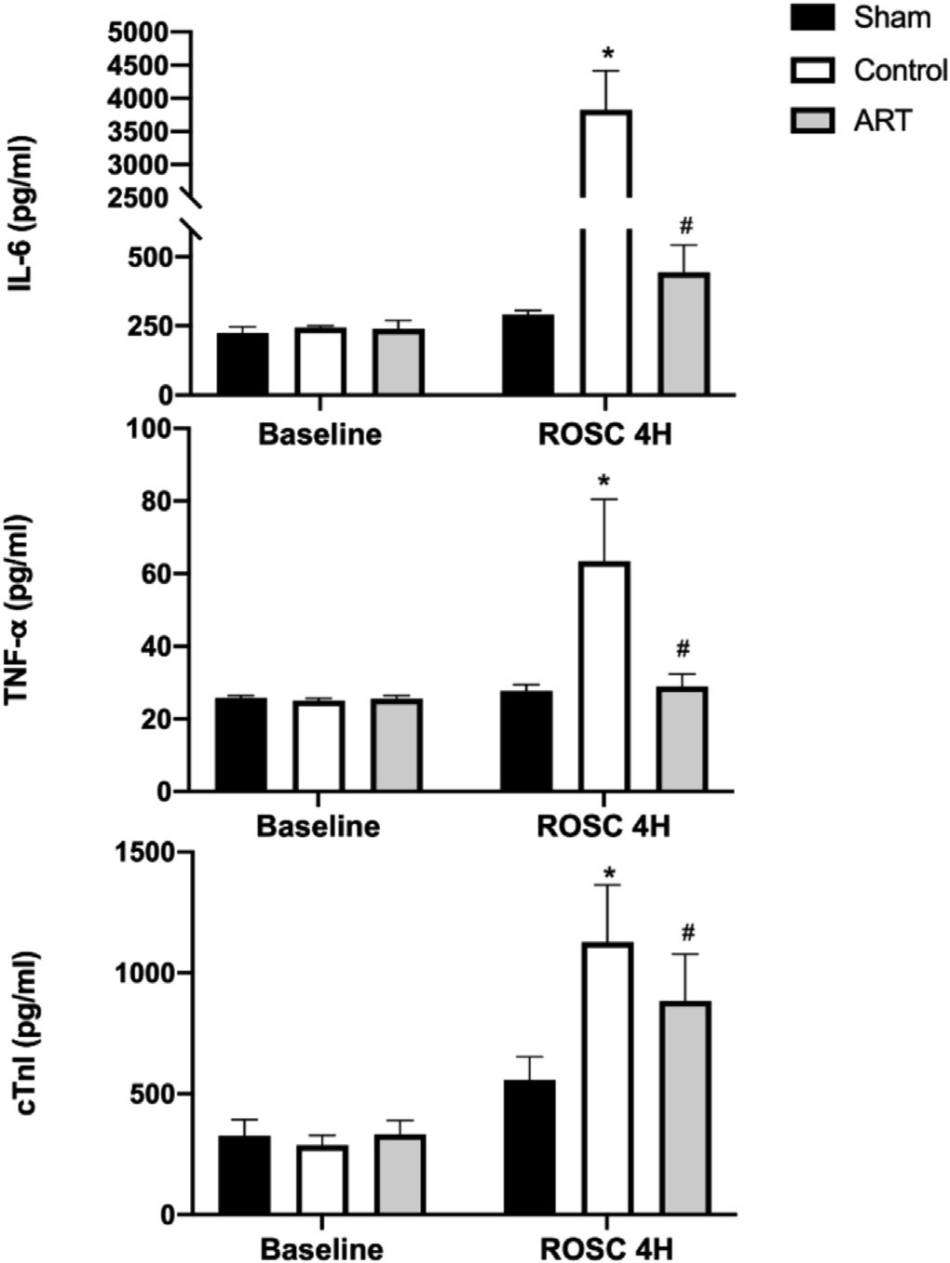
**Art attenuates post-resuscitation IL-6, TNF-α, and cTnI levels**. Interleukin 6 (IL-6), tumor necrosis factor-α (TNF-α) and Cardiac Troponin I (cTnI) levels in serum were measured with ELISA kit. H, hour; ROSC, Return of spontaneous circulation. Sham, surgical sham group without cardiac arrest; Control, CA/CPR + vehicle non-survival group; ART, CA/CPR + Art non-survival group. Data were presented as mean ± SD (*n* = 6 in each group). All *p < 0.0001 versus Sham, all ^#^*p* < 0.0001 versus Control.

Four hours after ROSC, IL-6, TNF-α and cTnI were significantly elevated in both the Control and ART groups compared to the Sham group. However, the concentrations of IL-6, TNF-α, and cTnI were significantly lower in the ART group compared to the Control group at 4 h post-ROSC (Fig. 5, Supplementary Table S2), indicating that Art reduces inflammatory and myocardial injury markers.

### Art attenuates oxidative stress-induced tissue damage

**Fig. 6:**
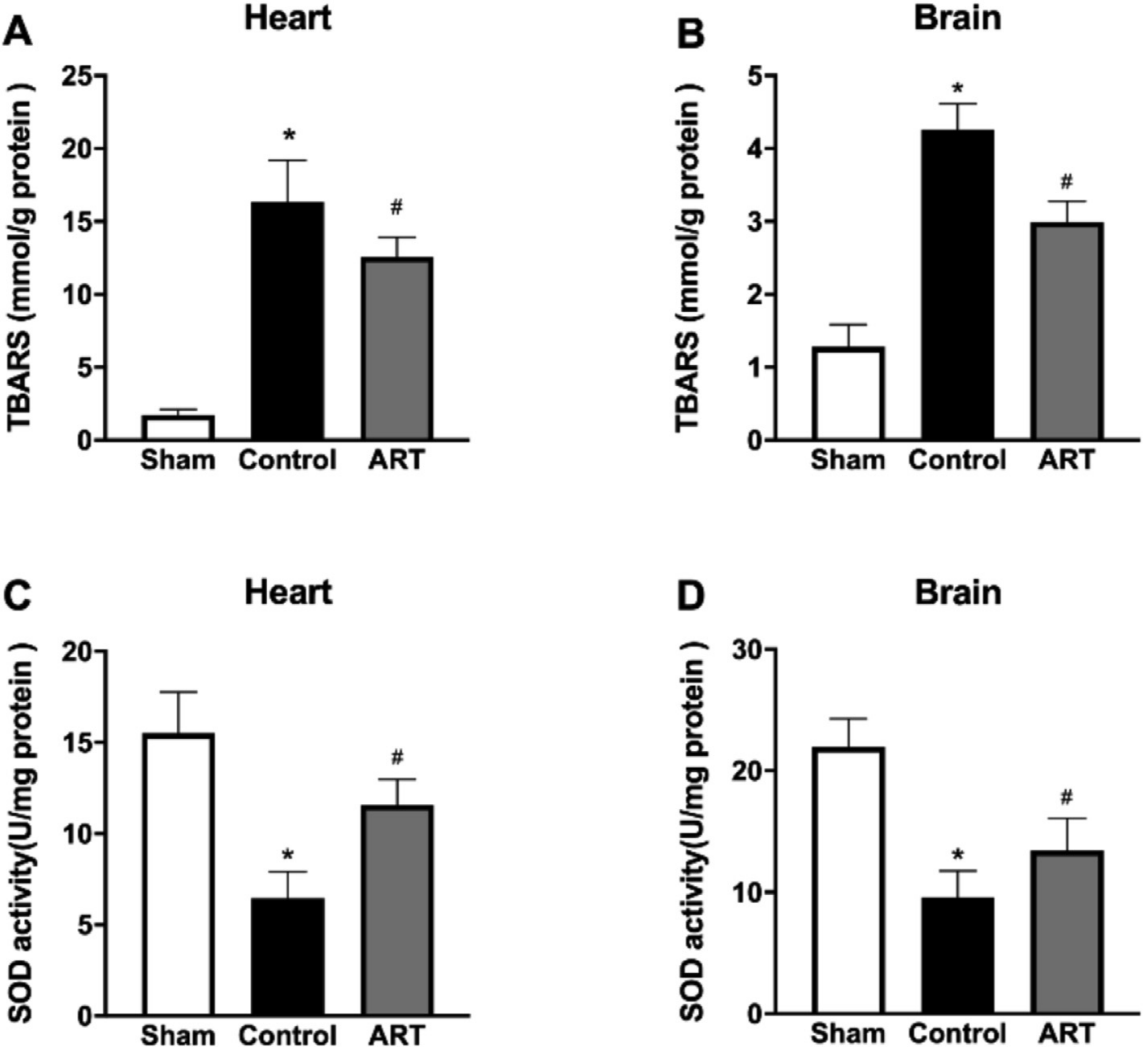
**Cardio- and neuro-protective effects of Art on post-resuscitation oxidative stress**. A. Effects of Art on TBARS concentrations in heart after post-resuscitation. B. Effects of Art on TBARS concentrations in brain after post-resuscitation. C. Effects of Art on SOD activities in heart after post-resuscitation. D. Effects of Art on SOD activities in brain after post-resuscitation. Superoxide dismutase (SOD) activity was determined using SOD Assay Kit, Thiobarbituric acid reacting level was measured using TBARS Assay Kit. Sham, surgical sham group without cardiac arrest; Control, CA/CPR + vehicle non-survival group; ART, CA/CPR + Art non-survival group; Data were presented as mean ± SD (*n* = 6 in each group). All *p < 0.0001 versus Sham; A: ^#^*p* < 0.0075 versus Control; B: ^#^*p* < 0.0001 versus Control; C: ^#^*p* = 0.0004 versus Control; D: ^#^*p* = 0.0336 versus Control.

Tissue homogenates from the heart and brain showed significantly higher TBARS levels in both the Control and ART groups compared to the Sham group (Fig. 6A, Supplementary Table S2), indicating increased oxidative damage. Additionally, SOD activity in heart and brain tissues was significantly depressed in both treated groups compared to the Sham group (Fig. 6A, D; Supplementary Table S2). Importantly, TBARS levels were significantly lower in Art-treated animals compared to the Control group (Fig. 6A, B; Supplementary Table S2). Furthermore, Art treatment increased total SOD activity in tissue homogenates (Fig. 6C, D, Supplementary Table S2), suggesting that Art alleviates oxidative stress damage.

### Art attenuates resuscitation-induced 4-HNE accumulation and pp38 activation

**Fig. 7:**
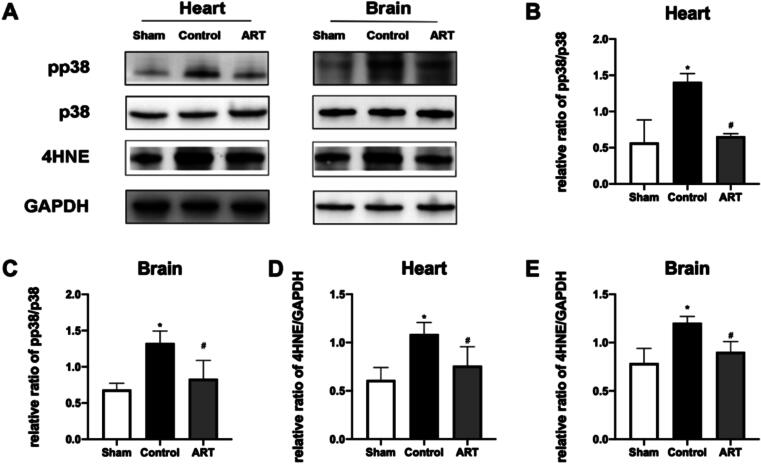
**Art attenuates resuscitation-induced 4-HNE accumulation and pp38 activation**. A. Representative protein bands of pp38, p38, 4-HNE proteins and GAPDH. B. Quantification of the relative pp38/p38 Ratio in heart tissue. C. Quantification of the relative pp38/p38 Ratio in brain tissue. D. Quantification of the expression levels of 4-HNE proteins in heart tissue. E. Quantification of the expression levels of 4-HNE proteins in brain tissue. Expressions of pp38, p38, 4-HNE proteins in the heart and brain were evaluated by western blotting at 64 h post-ROSC. Band intensities were analyzed by using ImageJ software and normalized to GAPDH. pp38, phosphorylation of p38; 4-HNE, 4-hydroxynonena; GAPDH, glyceraldehyde-3-phosphate dehydrogenase; Sham, surgical sham group without cardiac arrest; Control, CA/CPR + vehicle non-survival group; ART, CA/CPR + Art non-survival group. Data were presented as mean ± SD (*n* = 6 in each group). B: *p = 0.0044 versus Sham, ^#^*p* = 0.0076 versus Control; C: *p = 0.0121 versus Sham, ^#^*p* = 0.0376 versus Control; D: *p = 0.0002 versus Sham, ^#^*p* = 0.0056 versus Control; E: *p = 0.0098 versus Sham, ^#^*p* = 0.00403 versus Control.

After observing elevated 4-HNE levels in cardiac and brain tissues in a CA/CPR of rat model (Fig. 7D, E; Supplementary Table S2), we further examined the changes of p38 MAPK activation. At 4 h post-resuscitation, the protein levels of pp38 in both heart and brain were significantly increased compared with the sham group (Fig. 7B, C; Supplementary Table S2). Art treatment markedly attenuated these changes, significantly reducing the content of 4-HNE and p-p38 in cardiac and brain tissues compared with vehicle-treated CA/CPR rats (Fig. 7B–E, Supplementary Table S2).

## Discussions

This study demonstrated the effectiveness of Art on post-resuscitation myocardial and neurological function in a rat model of CA/CPR. Art significantly improved sublingual microcirculation, and increased 72-h survival after ROSC. These beneficial effects are closely associated with the dual inhibitory actions of Art on oxidative stress and systemic inflammation.

Myocardial injuries and microcirculatory derangements following cardiac arrest are major causes of early mortality and are strongly associated with poor outcome after resuscitation, primarily driven by I/R injury.^28^ Among the affected tissues, the endothelium is particularly susceptible to I/R damage, which results in increased vascular permeability, a hypercoagulable state, vasoconstriction, and local inflammation.^5^ Previous studies have shown that, despite restoration of macro-hemodynamics after ROSC, the microcirculation often remains heterogeneous, with capillary obstruction and sluggish flow, contributing to tissue hypoxia and multiple organ dysfunction. In the CA/CPR rat model, we observed significant deterioration of EF, CO, myocardial injury biomarkers, NDS, and sublingual microcirculation following resuscitation.

Previous studies have reported that Art attenuates I/R injury through the inhibition of mitochondrial autophagy, oxidative stress, and inflammatory responses.^20^, ^29^ Wang et al. demonstrated that Art alleviates cerebral I/R injury in middle cerebral artery occlusion rats by suppressing FUNDC1-mediated excessive mitophagy, thereby reducing infarct size, preserving neurological function, and improving memory performance.^20^ Consistent with these findings, the present study confirmed that the administration of Art after ROSC markedly improved cardiac function, neurological outcomes, and sublingual microcirculation, as well as prolonged 72-h survival.

Both experimental and clinical studies have shown that oxidative stress is markedly elevated after CA/CPR. A prospective study further reported that oxidative stress biomarkers in out-of-hospital cardiac arrest (OHCA) patients are closely correlated with neurological outcomes.^30^ SOD is a key enzymatic defense against superoxide radicals, whereas TBARS and 4-HNE reflect lipid peroxidation and membrane damage.^31^, ^32^ In our study, oxidative stress markers (TBARS, 4-HNE) were significantly elevated in the myocardium and brain tissues of CA/CPR rats. Treatment with Art restored SOD activity and reduced ROS-mediated lipid peroxidation, indicating that Art mitigates oxidative stress–induced injury, thereby improving post-resuscitation myocardial function and reducing neurological impairment. This finding aligns with previous studies. For instance, Lu et al. reported that Art demonstrates neuroprotective effects against cerebral I/R injury. This is achieved through the activation of the Nrf2 pathway and the ROS-dependent p38 MAPK pathways.^21^ Additionally, Liu et al. documented that Art significantly mitigates I/R-induced myocardial injury in mice. It does so by suppressing the increases in ROS, MDA, creatine kinase, and LDH levels.^29^

Excessive accumulation of ROS during I/R injury triggers cellular and molecular abnormalities, amplifies inflammatory responses, and activates cell death pathways.^33^

In the control group, plasma pro-inflammatory cytokines (IL-6 and TNF-α) were markedly elevated at 4 h after resuscitation. As potent inflammatory mediators, IL-6 and TNF-α stimulate the expression of endothelial adhesion molecules, thereby promoting leukocyte adhesion and transmigration, augmenting the release of inflammatory mediators into tissues, and driving systemic inflammation in the early post-resuscitation period; these processes further aggravate myocardial depression, endothelial dysfunction, and microcirculatory failure.^34^ Notably, the administration of Art significantly attenuated these inflammatory responses, reduced myocardial injury, and improved organ perfusion, as evidenced by reduced plasma IL-6 and TNF-α levels, decreased myocardial cTnI levels, and improved microcirculatory perfusion.

Of particular interest is the role of 4-HNE and p38 MAPK as key hubs linking oxidative stress and inflammation.^35^, ^36^, ^37^, ^38^ Accumulating evidence indicates that 4-HNE acts as an upstream stress signal to activate p38 MAPK,^39^, ^40^ and sustained p38 activation has been implicated in cardiomyocyte apoptosis, amplification of inflammatory responses, and functional deterioration during myocardial I/R injury.^36^, ^37^ This pathogenic role is supported by studies showing that pharmacological inhibition of p38 reduces infarct size and improves cardiac function in experimental models. In the present study, we observed an increase in pp38 levels in both the heart and brain following resuscitation. This activation was significantly attenuated by Art, paralleling the reduction in 4-HNE. The concurrent suppression of both 4-HNE and pp38 is consistent with the reported mechanism of 4-HNE-induced p38 activation, suggesting that the 4-HNE mediated activation of p38 MAPK signaling pathway may play an important role in linking oxidative stress to inflammatory responses after CA/CPR. Thus, by mitigating 4-HNE accumulation and subsequent p38 MAPK overactivation, Art may help disrupt this detrimental cycle.

Several limitations should be acknowledged. First, this study was conducted in healthy rats without comorbidities, whereas most OHCA patients present with underlying conditions, limiting the translational applicability. Second, only male rats were included in this study. This design choice may limit the generalizability of our findings to females. Future studies including both sexes are needed to evaluate potential sex-specific responses to Art after CA/CPR. Third, only one dose of Art, based on World Health Organization recommendations, was tested; future studies should investigate the dose–response and time–response relationships of Art in CA models. Fourth, no corresponding cell experiments were performed; the precise intracellular signaling networks by which 4-HNE-induced activation of p38 MAPK drives amplification of oxidative stress, production of inflammatory cytokines, and injury to cardiac and neuronal cells remain to be fully elucidated.

In conclusion, our findings suggest that Art exerts protective effects against oxidative stress and inflammatory injury in a rat model of CA/CPR. Post-resuscitation treatment with Art significantly improved myocardial and neurological functions, enhanced microcirculation, and prolonged survival. The cardiac cerebral protective effects of Art are likely attributable to its inhibition of I/R-induced oxidative and inflammatory cascades, and the 4-HNE mediated activation of p38 MAPK signaling pathway may represent an important mediator of Art’s protective actions. These results indicate that Art may represent a promising therapeutic option for protecting vital organs during and after cardiopulmonary resuscitation.

## Data sharing statement

The data sets used and/or analyzed during the current study are available from the corresponding author on reasonable request.

## CRediT authorship contribution statement

**Hui Li:** Writing – original draft, Investigation, Conceptualization. **Cheng Cheng:** Investigation, Conceptualization. **Lian Liang:** Investigation, Formal analysis. **Tao Jin:** Investigation, Formal analysis. **Guozhen Zhang:** Investigation. **Mary Ann Peberdy:** Supervision, Formal analysis. **Joseph P. Ornato:** Supervision, Formal analysis. **Wanchun Tang:** Writing – review & editing, Supervision. **Min Yang:** Writing – review & editing, Supervision.

## Ethics approval and consent to participate

This study was performed under a protocol (AD10001396, specific aim 21) approved by the Institutional Animal Care and Use Committee of Virginia Commonwealth University. All animal experiments were conducted in accordance with the National Institutes of Health *Guide for the Care and Use of Laboratory Animals* (National Research Council, 8th edition, 2011)

## Funding

This work was supported in part by the 10.13039/100020057Weil Family Foundation, California. The funding agencies had no role in study design, data collection or analysis.

## Declaration of competing interest

The authors declare that they have no competing interests.
