# Supplementary material for: Artesunate preserves post-resuscitation myocardial and neurologic function in a rat model of cardiac arrest and cardiopulmonary resuscitation
Source: Resusc Plus. 2025 Dec 30;27:101214. doi: 10.1016/j.resplu.2025.101214 (PMC12825069; doi:10.1016/j.resplu.2025.101214)
Supplement: Supplementary Data 1 [file mmc1.docx]

**Supplemental Materials**

**Figure S1. Images of long axis, long axis contains the heart and aorta wall and MPI flow**

A echocardiographic planes of long axis

B echocardiographic planes of long axis contains the heart and aorta wall

C Images of MPI flow

MPI, myocardial performance index;


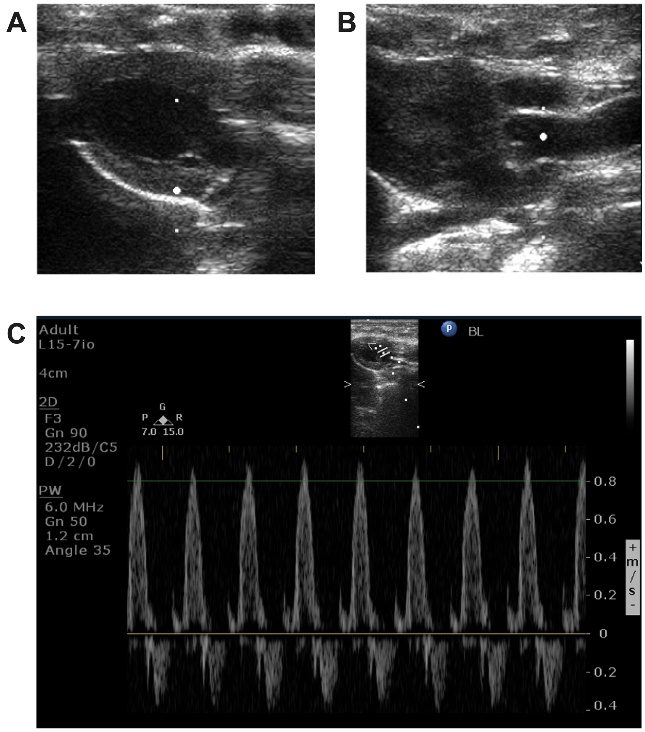


**Figure S2. Images of sublingual microcirculation**

Representative sublingual microcirculation images acquired using side-stream dark field imaging with a × 5 imaging, resulting an on-screen magnification of 276 ×. Sham, surgical sham group without cardiac arrest; Control, CA/CPR + vehicle non- survival group; ART, CA/CPR + Art non- survival group; Scale bar = 20 µm.


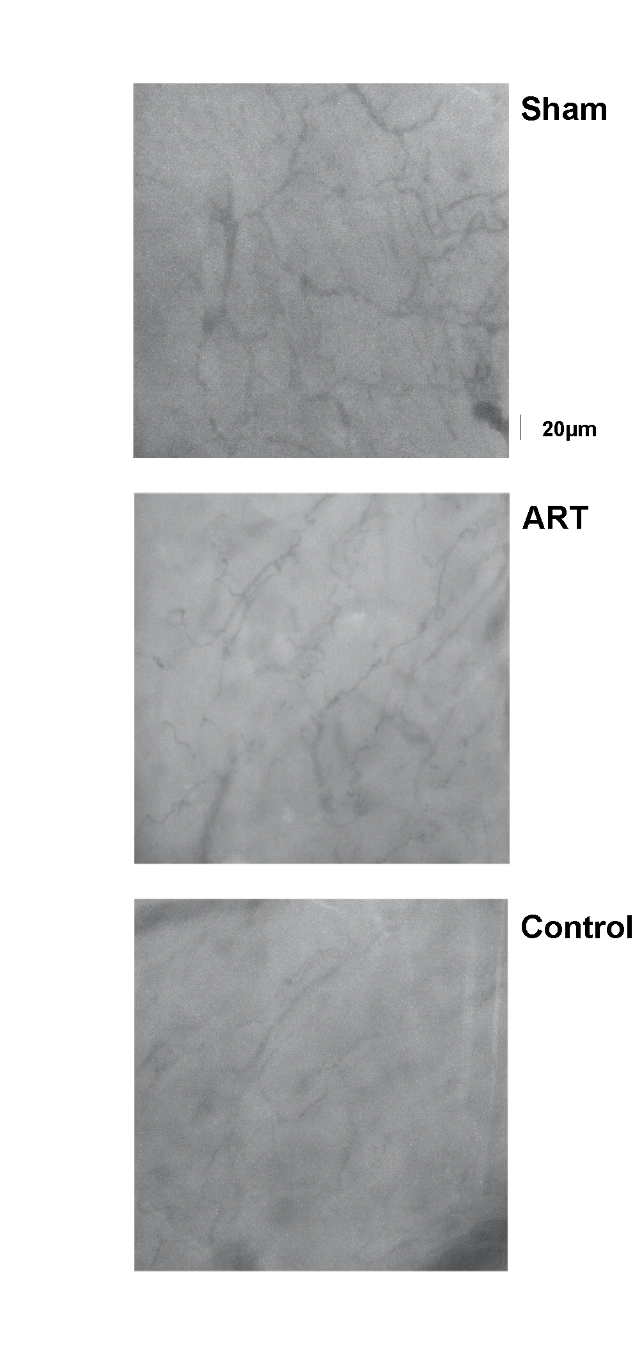


**Table S1. Baseline characteristics and post-CA/CPR measurements**

MAP, mean aortic pressures; PC, Precordial chest; ETCO_2_, end-tidal carbon dioxide; EF, Ejection fraction; CO, Cardiac output; MPI, Myocardial performance index; MFI, Microcirculatory flow index; PVD, Perfused vessel density. Data were presented as mean ± SD (n = 6 in each group).

| Table S1. Baseline characteristics and post-CA/CPR measurements | | | | | | |
| --- | --- | --- | --- | --- | --- | --- |
| Variables | Sham | CA/CPR + vehicle | | CA/CPR + Art | *p* | |
|  |  | Non-survival | Survival | Non-survival | Survival |  |
|  | (n=6) | (n=6) | (n=6) | (n=6) | (n=6) |  |
| Body weights (g) | $493\pm23.9$ | $501\pm19.9$ | $500\pm14.7$ | $491\pm18.7$ | $485\pm15.7$ | 0.501 |
| Heart rate (bpm) | $376\pm15$ | $372\pm32$ | $371\pm22$ | $397\pm27$ | $387\pm24$ | 0.365 |
| MAP (mmHg) | $131\pm11$ | $133\pm12$ | $128\pm10$ | $138\pm7$ | $137\pm16$ | 0.418 |
| Temperature (℃) | $36.9\pm0.4$ | $36.6\pm0.5$ | $36.8\pm0.5$ | $36.6\pm0.6$ | $36.7\pm0.5$ | 0.832 |
| PC depth (mm) | … | $13\pm1$ | $13\pm1$ | $12\pm2$ | $12\pm1$ | 0.093 |
| N0. of shocks | … | $1.3\pm0.5$ | $1.2\pm0.4$ | $1.3\pm0.8$ | $1.0\pm0.6$ | 0.701 |
| ETCO_2_ (mmHg) | $35.3\pm4.3$ | $37.2\pm5.2$ | $37.1\pm3.9$ | $36.4\pm6.2$ | $36.2\pm5.2$ | 0.959 |
| EF (%) | $73\pm6$ | $69\pm5$ | $70\pm5$ | $70\pm4$ | $71\pm5$ | 0.793 |
| CO (ml/min) | $125.8\pm7.7$ | $124\pm8.1$ | $125.4\pm10.5$ | $123.6\pm11.5$ | $129.3\pm12.3$ | 0.794 |
| MPI | $0.65\pm0.04$ | $0.67\pm0.05$ | $0.67\pm0.07$ | $0.69\pm0.04$ | $0.68\pm0.05$ | 0.580 |
| MFI | 3 | 3 | 3 | 3 | 3 | 1 |
| PVD | $5.84\pm0.37$ | $5.75\pm0.40$ | $5.89\pm0.37$ | 6.04$\pm0.22$ | $5.77\pm0.32$ | 0.572 |

**Table S2. Functional, microcirculatory, and biochemical parameters in the groups**

CA: Cardiac arrest; CPR, cardiopulmonary resuscitation. Sham, surgical sham group without cardiac arrest; H, hour; MAP, mean aortic pressures; PC, Precordial chest; ETCO2, end-tidal carbon dioxide; EF, Ejection fraction; CO, Cardiac output; MPI, Myocardial performance index; MFI, Microcirculatory flow index; PVD, Perfused vessel density; IL-6: Plasma levels of interleukin-6; TNF-α: tumor necrosis factor-α; cTnI: cardiac troponin I; TBARS: Thiobarbituric acid reactive species; SOD: Superoxide dismutases; CNS: Central nervous system; 4-HNE: 4-hydroxy-2-nonenal; MAPK: Mitogen activated protein kinase; pp38: phosphorylated p38. Survival rate data were presented as survival animal counts to total animal number of each group, other data were presented as mean ± SD (n = 6 in each group).

| Table S2. Functional, microcirculatory, and biochemical parameters in the groups | | | | | |
| --- | --- | --- | --- | --- | --- |
| Variables | Sham  (n=6) | CA/CPR + vehicle | | CA/CPR + Art | |
|  |  | Non-survival  (n=6) | Survival  (n=6) | Non-survival  (n=6) | Survival  (n=6) |
| 1H EF (%) | $69.42\pm2.61$ | $45.60\pm5.03$ | $\ldots$ | $48.67\pm4.60$ | $\ldots$ |
| 2H EF (%) | $69.88\pm2.11$ | $49.43\pm3.75$ | $\ldots$ | $60.10\pm2.53$ | $\ldots$ |
| 3H EF (%) | $70.97\pm1.34$ | $51.03\pm1.69$ | $\ldots$ | $61.75\pm3.75$ | $\ldots$ |
| 4H EF (%) | $68.07\pm2.14$ | $51.50\pm3.19$ | $\ldots$ | $61.90\pm1.33$ | $\ldots$ |
| 1H CO (ml/min) | $122.83\pm7.99$ | $60.95\pm5.55$ | $\ldots$ | $73.15\pm9.57$ | $\ldots$ |
| 2H CO (ml/min) | $125.00\pm5.73$ | $72.12\pm9.70$ | $\ldots$ | $107.07\pm20.59$ | $\ldots$ |
| 3H CO (ml/min) | $123.83\pm2.48$ | $84.48\pm2.76$ | $\ldots$ | $108.38\pm18.75$ | $\ldots$ |
| 4H CO (ml/min) | $123.50\pm7.06$ | $77.58\pm16.11$ | $\ldots$ | $111.62\pm14.95$ | $\ldots$ |
| 1H MPI | $0.66\pm0.05$ | $1.19\pm0.09$ | $\ldots$ | $1.00\pm0.07$ | $\ldots$ |
| 2H MPI | $0.66\pm0.04$ | $1.18\pm0.05$ | $\ldots$ | $0.80\pm0.09$ | $\ldots$ |
| 3H MPI | $0.66\pm0.05$ | $1.23\pm0.09$ | $\ldots$ | $0.81\pm0.13$ | $\ldots$ |
| 4H MPI | $0.58\pm0.18$ | $1.12\pm0.04$ | $\ldots$ | $0.83\pm0.12$ | $\ldots$ |
| 1H MFI | $2.89\pm0.10$ | $1.32\pm0.38$ | $\ldots$ | $2.04\pm0.22$ | $\ldots$ |
| 2H MFI | $2.90\pm0.06$ | $1.43\pm0.21$ | $\ldots$ | $2.11\pm0.31$ | $\ldots$ |
| 3H MFI | $2.89\pm0.10$ | $1.36\pm0.25$ | $\ldots$ | $2.13\pm0.20$ | $\ldots$ |
| 4H MFI | $2.93\pm0.10$ | $1.44\pm0.38$ | $\ldots$ | $2.19\pm0.27$ | $\ldots$ |
| 1H PVD | $5.76\pm0.15$ | $3.56\pm0.44$ | $\ldots$ | $4.75\pm0.48$ | $\ldots$ |
| 2H PVD | $5.68\pm0.29$ | $3.57\pm0.53$ | $\ldots$ | $4.71\pm0.40$ | $\ldots$ |
| 3H PVD | $5.71\pm0.48$ | $3.33\pm0.60$ | $\ldots$ | $4.82\pm0.44$ | $\ldots$ |
| 4H PVD | $5.66\pm0.30$ | $3.41\pm0.44$ | $\ldots$ | $4.93\pm0.26$ | $\ldots$ |
| Survival rate | | | | | |
| 24H | $\ldots$ | $\ldots$ | $4/6$ | $\ldots$ | $6/6$ |
| 48H | $\ldots$ | $\ldots$ | $2/6$ | $\ldots$ | $6/6$ |
| 72H | $\ldots$ | $\ldots$ | $2/6$ | $\ldots$ | $5/6$ |
| 24H NDS | $\ldots$ | $\ldots$ | $343\pm134$ | $\ldots$ | $126\pm49$ |
| 48H NDS | $\ldots$ | $\ldots$ | $375\pm194$ | $\ldots$ | $86\pm53$ |
| 72H NDS | $\ldots$ | $\ldots$ | $378\pm191$ | $\ldots$ | $136\pm183$ |
| IL-6 (pg/ml) | | | | | |
| Baseline | $225\pm21$ | $243\pm6$ | $\ldots$ | $239\pm31$ | $\ldots$ |
| 4H | $291\pm14$ | $3825\pm589$ | $\ldots$ | $444\pm98$ | $\ldots$ |
| TNF-α (pg/ml) | | | | | |
| Baseline | $25.72\pm0.65$ | $25.08\pm0.59$ | $\ldots$ | $25.58\pm0.86$ | $\ldots$ |
| 4H | $27.61\pm1.77$ | $61.37\pm17.08$ | $\ldots$ | $28.95\pm3.40$ | $\ldots$ |
| cTnI (pg/ml) | | | | | |
| Baseline | $325\pm67$ | $286\pm42$ | $\ldots$ | $331\pm58$ | $\ldots$ |
| 4H | $557\pm95$ | $1127\pm236$ | $\ldots$ | $885\pm193$ | $\ldots$ |
| TBARS (mmol/g protein) | | | | | |
| heart | $1.71\pm0.40$ | $16.35\pm2.85$ | $\ldots$ | $12.58\pm1.33$ | $\ldots$ |
| brain | $1.28\pm0.30$ | $4.26\pm0.35$ | $\ldots$ | $2.99\pm0.28$ | $\ldots$ |
| SOD (U/mg protein) | | | | | |
| heart | $15.51\pm2.26$ | $6.47\pm1.45$ | $\ldots$ | $11.58\pm1.39$ | $\ldots$ |
| brain | $21.96\pm2.31$ | $9.58\pm2.17$ | $\ldots$ | $13.44\pm2.64$ | $\ldots$ |
| pp38/p38 | | | | | |
| heart | $0.57\pm0.31$ | $1.41\pm0.11$ | $\ldots$ | $0.66\pm0.03$ | $\ldots$ |
| brain | $0.69\pm0.09$ | $1.33\pm0.17$ | $\ldots$ | $0.83\pm0.25$ | $\ldots$ |
| 4-HNE/GAPDH | | | | | |
| heart | $0.61\pm0.13$ | $1.09\pm0.12$ | $\ldots$ | $0.76\pm0.20$ | $\ldots$ |
| brain | $0.79\pm0.15$ | $1.21\pm0.07$ | $\ldots$ | $0.90\pm0.10$ | $\ldots$ |
